# The impact of circulation in a heart–lung machine on function and survival characteristics of red blood cells

**DOI:** 10.1111/aor.13682

**Published:** 2020-04-03

**Authors:** Joames Freitas Leal, Harry Vermeer, Dan Lazari, Leen van Garsse, Roland Brock, Merel Adjobo‐Hermans, Giel Bosman

**Affiliations:** ^1^ Department of Biochemistry Radboudumc Nijmegen The Netherlands; ^2^ Department of Cardiothoracic Surgery Radboud University Medical Center Nijmegen The Netherlands

**Keywords:** aging, extracorporeal circulation, heart–lung machine, red blood cell

## Abstract

Extracorporeal circulation is accompanied by changes in red blood cell morphology and structural integrity that affect cell function and survival, and thereby may contribute to the various side effects of heart–lung machine‐assisted surgery. Our main objectives were to determine the effect of circulation of red blood cells in a stand‐alone extracorporeal circuit on several parameters that are known to be affected by, as well as contribute to red blood cell aging. As a source of RBCs, we employed blood bank storage units of different ages. In order to assess the relevance of our in vitro observations for the characterization of extracorporal circulation technology, we compared these changes in those of patients undergoing extracorporeal circulation‐assisted cardiac surgery. Our results show that circulation in a heart–lung machine is accompanied by changes in red blood cell volume, an increase in osmotic fragility, changes in deformability and aggregation behavior, and alterations in the exposure of phosphatidylserine and in microvesicle generation. RBCs from 1‐week‐old concentrates showed the highest similarities with the in vivo situation. These changes in key characteristics of the red blood cell aging process likely increase the susceptibility of red blood cells to the various mechanical, osmotic, and immunological stress conditions encountered during and after surgery in the patient’s circulation, and thereby contribute to the side effects of surgery. Thus, aging‐related parameters in red blood cell structure and function provide a foundation for the validation and improvement of extracorporeal circulation technology.

## 1 INTRODUCTION

Maintenance of oxygen supply by extracorporeal circulation (EC) creates various nonphysiological conditions that contribute to postoperative complications such as thrombosis, intravascular hemolysis, and inflammation.^1^, ^2^, ^3^ These complications affect oxygen delivery,^4^ induce anemia,^3^, ^5^ and may contribute to short‐term as well as long‐term defects in hemostasis^3^, ^4^ and to various neurological problems.^5^, ^6^ Changes in red blood cell (RBC) function and homeostasis are likely to contribute to these symptoms. During EC, the RBCs experience mechanical stress induced by the tubing material, the pump and the oxygenator, which may lead to changes in deformability, membrane composition, and organization that affect tissue oxygenation and promote inflammation and coagulation.^7^

So far, determination of the effect of EC of RBCs has mostly been restricted to end‐stage parameters such as hemolysis and thrombosis, and measuring the degree of hemolysis is the only mandatory condition in the testing of device safety and hemocompatibility.^8^ Relatively few studies have addressed the effect of EC on the processes regulating fundamental characteristics of RBCs.^3^, ^9^, ^10^ Therefore, we investigated the effect of circulation in a heart–lung machine on physiologically relevant parameters of RBC structure, function, and survival.^11^, ^12^, ^13^, ^14^, ^15^, ^16^, ^17^ In order to distinguish the EC‐induced changes from those influenced by the patients’ circulation,^10^, ^12^ we compared the data obtained from a stand‐alone extracorporeal circuit with those obtained during EC‐assisted cardiac surgery. Furthermore, we addressed the dependence of these changes on the age of RBC storage units, to identify whether a certain age mimics best the changes occurring in vivo.

Our results show that (1) changes in RBC morphology, susceptibility to osmotic stress, deformability, aggregation, and the appearance of removal signals such as phosphatidylserine occur during circulation in a heart–lung machine, both in vitro in a stand‐alone extracorporeal circuit and during surgery; (2) the extent and kinetics of these changes are modified by the storage time in the blood bank. From these data, we conclude that the circulation of RBCs with a short blood bank storage time in a stand‐alone extracorporeal circuit constitutes a clinically relevant system for the development, improvement, and quality control of extracorporeal circuit technology.

## 2 MATERIALS AND METHODS

### 2.1 Red blood cell concentrates

Stored RBCs were purchased as standard RBC concentrates from the regional blood bank (Sanquin Bank South East Region, Nijmegen, The Netherlands).^11^ At each storage time point, RBCs from three concentrates were pooled in order to reduce interindividual variability,^10^, ^11^, ^13^ and washed three times using Ringer's solution (125 mM NaCl, 5 mM KCl, 1 mM MgSO_4_, 2.5 mM CaCl_2_, 5 mM glucose, 32 mM HEPES, pH 7.4) by repeated centrifugation (10 minutes, 1500 x *g*).

### 2.2 Stand‐alone extracorporeal circuit

The extracorporeal circuit was designed to mimic a clinical cardiopulmonary bypass circuit and consisted of a hard‐shell venous reservoir (Inspire HVR, LivaNova, Mirandola, Italy), 3/8 inch tubing with a phosphorylcholine‐based coating (LivaNova), a roller pump, an oxygenator (Inspire 6, LivaNova), an arterial filter (Pall AL6, Terumo Europe, Leuven, Belgium), and an arterial cannula (EOPA 22 Fr, Medtronic, Heerlen, The Netherlands). The priming fluid, identical to that used during surgery, was composed of 1000 mL of gelofusin (40 g/L, B. Braun, Melsungen, Germany), 50 mL of albumin solution (Albuman 200 g/L, Sanquin Plasma Products, Amsterdam, The Netherlands), 3750 IE unfractionated heparin (LEO Pharma, Amsterdam, The Netherlands), 25 mL of sodium bicarbonate (84 g/L, B. Braun), 5 mL of calcium gluconate (100 mg/mL, B. Braun), and 100 mL of mannitol (150 g/L, Baxter Nederland, Utrecht, The Netherlands). Before the start of the experiments, RBCs were suspended in priming fluid and the volume of priming fluid in the heart–lung machine was adjusted in order to achieve a starting hematocrit of 25%.

Experimental conditions were comparable to those maintained during cardiac surgery, employing a flow rate of 4.5 L/min, a postoxygenator pressure of 178 mm Hg (± 2 mm Hg) generated by an occluder between the arterial filter and arterial cannula, a fraction of inspired oxygen of 21%, a CO_2_ flow of 4 mL/min, a sweep gas flow of 1000 mL/min, and a fluid temperature of 36.7 ± 0.1°C.

### 2.3 Cardiac surgery

The study protocol was approved by the local ethics committee (Commissie Mensgebonden Onderzoek regio Arnhem‐Nijmegen, The Netherlands; file number 2018‐4421) and informed consent was obtained from all participants. The same semi‐closed extracorporeal circuits were used for all patients. These circuits included a soft‐shell venous reservoir (VBR 1900, Getinge, Hilversum, The Netherlands), a centrifugal pump (Revolution, LivaNova), a hollow fiber oxygenator (Quadrox‐i adult, Getinge), an arterial filter (PALL AL6), a hard‐shell cardiotomy reservoir (VHK 71000, Getinge), and coated tubing (Bioline, Getinge). The extracorporeal circuits contained 1200 mL of priming fluid, with a composition as described above for the stand‐alone experiments. Standard cannulas were used: arterial 22 Fr DLP (Medtronic) and venous ual tage 36/46 Fr (Edwards, Dilbeek, Belgium).

Anesthesia was performed according to local guidelines. After premedication with oral paracetamol and midazolam, anesthesia was induced with sufentanil, midazolam, propofol, rocuronium, and maintained with sufentanil and midazolam. Just before and during the procedure, patients were given tranexamic acid with a bolus of 1000 mg in 15 minutes and continuously 400 mg/h. Patients were treated with the antibiotic cefazolin of 2000 mg (Eurocept, Ankeveen, The Netherlands) administered 60‐15 minutes before incision, which was repeated after 4 hours of surgery or when more than 500 mL of cell salvage product was returned to the patient.

Surgery was performed according to local guidelines. In brief, patients were heparinized using an initial dose of 300 IU/kg unfractionated heparin (LEO Pharma), and during surgery, the activated clotting time was maintained at >480 seconds. Patients were mildly cooled (>35°C nasal temperature) and rewarmed before weaning from the heart–lung machine (nasal temperature >36.5°C, peripheral temperature >35°C). Targeted flow rates during cardiopulmonary bypass were 2.6 L min m^2^, yielding a venous saturation of 70%‐80%. The average cardiopulmonary bypass time was 87 ± 31 minutes (*N* = 5). When needed, sevoflurane and noradrenalin were administered to maintain mean arterial blood pressure of 50‐60 mm Hg. Hyperkalemic warm blood cardioplegia according to Calafiori was used to arrest the heart and was repeated every 15‐25 minutes. Blood from the operative field was processed with an autotransfusion device. Protamine hydrochloride (Meda Pharma, Amstelveen, The Netherlands) in a 1:1 ratio with administered heparin was employed to neutralize residual heparin activity following surgery. After weaning from the heart–lung machine, residual blood in the extracorporeal circuit was processed by an autotransfusion system (XTRA, LivaNova) and immediately returned to the patient. Five patients (four males) were included in this pilot study. Their relevant demographics (average ± standard deviation) were body surface area 2.07 ± 0.11 m^2^, age 66 ± 7 years, cardiopulmonary bypass time 85 ± 33 minutes and aortic clamp time 45 ± 12 minutes. The total volume of the autotransfusion blood that was returned to the patients was 533 ± 89 mL, indicating the absence of large intraoperative differences in blood loss.

### 2.4 RBC analysis

About 5 mL of blood samples was taken before starting the heart–lung machine and subsequently every 30 minutes during extracorporeal circulation, and after extracorporeal circulation before patients left the operation room. RBCs were isolated by centrifugation (5 minutes, 400 x *g*). The degree of hemolysis was estimated by measuring the absorbance of the supernatant at 415 nm.

Semi‐quantitative microscopic analysis and cell morphology classification of RBCs into discocytes, echinocytes, ovalocytes, and stomatocytes was performed as described before.^14^

The percentage of phosphatidylserine (PS)‐exposing RBCs was determined using Annexin V, essentially as described before.^13^

Deformability and aggregation were measured using a laser‐assisted optical rotational cell analyzer (Lorrca MaxSis, Mechatronics, The Zwaag, The Netherlands) as described previously.^15^ Osmotic fragility was assessed by measuring the free hemoglobin concentration at 415 nm after incubation of the RBCs in various NaCl concentrations (150, 125, 100, 75, 50, and 0 mM).^16^

Microvesicles were isolated from the supernatants obtained after centrifugation of RBC samples (5 minutes, 400 x *g*) and analyzed by flow cytometry as described before.^17^

### 2.5 Statistical analysis

Differences between two groups were determined using a paired *t* test. Differences between three groups and time points were determined by repeated one‐way ANOVA in combination with Tukey’s post‐test. Two‐sided *P* values were used to determine statistical significance when *P* < .05.

## 3 RESULTS

**Figure 1:**
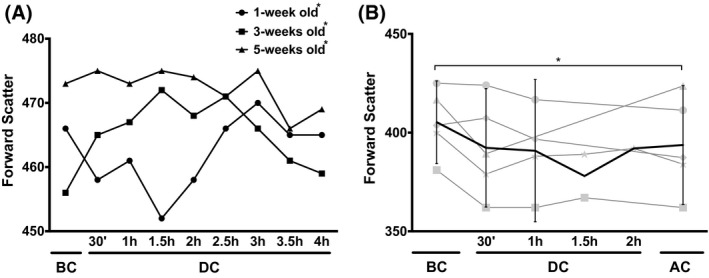
RBC forward scatter before, during, and after extracorporeal circulation. A, forward scatter of 1‐, 3‐, and 5‐weeks‐old RBCs during circulation in the heart–lung machine. For each storage period, RBCs of three different donors were pooled, as described in Materials and Methods. Data points are the mean of three independent measurements of three pooled RBC concentrates (SD not shown; ≤14 FSC units for all data points; 95% CI: 1‐week old: 458.1‐466.6; 3‐weeks old: 460.9‐469.1; 5‐weeks old: 469.9‐474.7); B, forward scatter of RBCs from five patients who underwent coronary bypass surgery with extracorporeal circulation + confidence limits (95% CI). Forward scatter is expressed as the median of the height of the forward scatter peak obtained by flow cytometry. All differences in the forward scatter between BC and 4 hours in panel A, and the differences between BC and AC in panel B are statistically significant (*P* < .05), as are the differences between 1‐week‐old, 3‐weeks‐old, and 5‐weeks‐old RBCs. BC, before extracorporeal circulation; DC, during extracorporeal circulation; AC, after extracorporeal circulation. For details see Materials and Methods; *, *P* < .05

We aimed at a deeper understanding of the impact of extracorporeal assisted circulation on functional RBC parameters. Since blood bank storage units provide a more accessible source of RBCs than surgical interventions, we compared the impact of extracorporeal circulation on RBCs of both sources. Aging in vitro during blood bank storage has been recognized as a critical parameter on a number of RBC‐associated parameters, such as stress‐induced phosphatidylserine exposure.^11^, ^13^ Therefore, we included blood bank storage units of different ages to assess whether RBCs of any age mimicked best the in vivo situation. In the stand‐alone extracorporeal circuit experiments, 1‐week‐old RBCs showed an initial, transient decrease in forward scatter, followed by an increase beyond the values obtained during the first 2 hours. Three‐weeks‐old RBCs showed a steady increase up to 2.5 hours of circulation, followed by a small decrease. Five‐weeks‐old RBCs showed a slight decrease in forward scatter throughout the circulation time (Figure 1A).

For the in vivo analysis, we collected RBCs from five patients scheduled for elective coronary artery bypass grafting, using both the left internal thoracic artery and a saphenous vena as conduits. To minimize any risk associated with additional blood sampling, we applied the following exclusion criteria: a body surface area <2.0 m^2^, grafting conduits other than the left internal thoracic artery and saphenous vein, age >75 year, preoperative hematocrit <30%. In order to avoid heterogeneity and interference with our measurements, we also excluded patients with normovolemic hemodilution and pre‐ or peroperative RBC transfusion.

The RBCs from patients undergoing EC during cardiac surgery showed changes that were similar to those of 1 week‐stored RBCs, that is, an initial decrease in forward scatter followed by an increase toward the starting value (Figure 1B).

**Figure 2:**
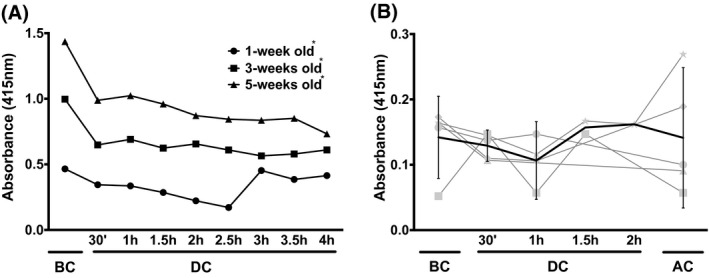
Osmotic fragility of RBCs during extracorporeal circulation. A, osmotic fragility of stored RBCs during circulation in a stand‐alone extracorporeal circuit, measured as the absorption of cell‐free hemoglobin at 415 nm after the incubation of RBCs in 100 mM NaCl. All data are the mean of three independent measurements of three pooled RBC concentrates (SD ≤ 0.007 absorbance units; 95% CI: 1‐week old: 0.264‐0.419; 3‐weeks old: 0.564‐0.764; 5‐weeks old: 0.793‐1.106); B, osmotic fragility of RBCs from five patients who underwent cardiac bypass surgery with extracorporeal circulation + confidence limits (95% CI), as described in the legend of Figure 1 and Materials and Methods. All data were extracted from RBC fragility curves (supplemental Figure 2). In panel A, the values of 1‐week, 3‐weeks, and 5‐weeks‐old RBC are significantly different from each other for all time points (*P* < .001). Abbreviations are as described in the legend to Figure 1

The ability to withstand hypo‐osmotic conditions decreased with storage time (Figure 2A), as reported before.^11^, ^16^, ^19^ In the stand‐alone extracorporeal circuit, the ability to withstand osmotic changes increased during the first hours of circulation, especially for the 1‐week‐old RBCs (Figure 2A). The RBCs from patients undergoing surgery showed small, nonuniform changes in osmotic fragility (Figure 2B; Figure S1 in Supplemental Data).

**Figure 3:**
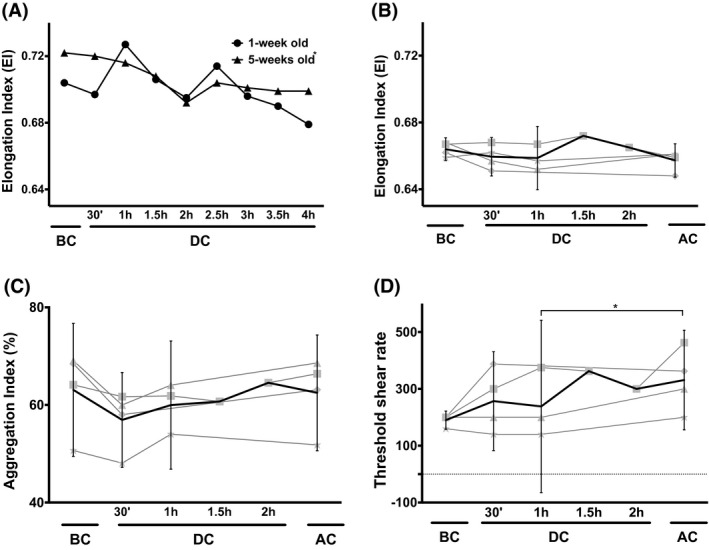
The effect of storage and circulation time on RBC deformability and aggregation. A, Maximal deformability, expressed as elongation index, of 1‐week and 5‐weeks‐old RBCs during circulation in a heart–lung machine. The standard deviation of all values was less than 0.01 EI units (95% CI: 1‐week old: 0.690‐0.711; 5‐weeks old: 0.698‐0.714); B, Deformability of the patients’ RBCs during extracorporeal circulation‐assisted cardiac surgery + confidence interval (95% CI); C, Aggregation Index of patients’ RBCs + confidence interval (95% CI); D, the minimal shear rate needed to prevent aggregation of the patients’ RBCs + confidence limits (95% CI). The data are the mean of four donors. In panel A, the values of 5‐weeks old RBCs are significantly different from each other for all time points, as are the differences in threshold shear rate between 1 hour of EC‐assisted surgery and after surgery in panel D (*, *P* < .01). Abbreviations are as described in the legend to Figure 1

Circulation in the stand‐alone extracorporeal circuit induced a decrease in the deformability of all blood bank RBCs (Figure 3A, Figure S1 in Supplemental Data), whereas the deformability of the patients’ RBCs did not change during surgery (Figure 3B). Moreover, both the aggregation index, a parameter for the tendency to form aggregates, and the threshold shear stress, an indication for the strength of the aggregates, increased to a stable level during EC‐assisted surgery (Figures 3C,D).

**Figure 4:**
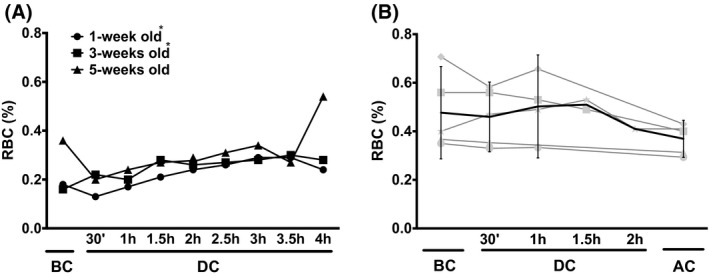
Exposure of phosphatidylserine during circulation in a heart–lung machine. A, Percentage of PS‐exposing RBCs in 1‐week, 3‐weeks, and 5‐weeks‐old RBC concentrates during circulation in the heart–lung machine. For each storage period, RBCs of three different donors were pooled, as described in Materials and Methods. The data represent the mean value of three representative measurements of at least 200 000 cells/measurement. (SD ≤ 0.02 %; 95% CI: 1‐week old: 0.180‐0.265; 3‐weeks old: 0.214‐0.285; 5‐weeks old: 0.238‐0.388); B, fraction of PS‐exposing RBC of five patients who underwent extracorporeal circulation‐assisted cardiac bypass surgery + confidence limits (95% CI). In panel A, the values of 1‐week and 3‐weeks‐old RBC are significantly different from each other for all time points (*, *P* < .05). The values of the patients’ RBCs (panel B) did not differ significantly from each other. Abbreviations are as described in the legend to Figure 1

The fraction of PS‐exposing RBCs from the blood bank increased during circulation in the stand‐alone extracorporeal circuit followed by a small decrease, with the exception of the 5‐weeks‐old RBCs (Figure 4A). The patients’ RBCs showed a similar pattern during surgery, that is, an initial increase followed by a small decrease (Figure 4B).

**Figure 5:**
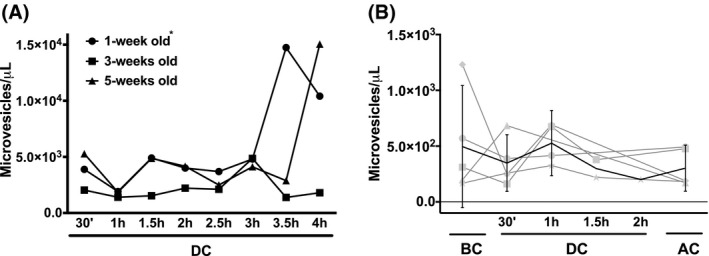
Microvesicle generation of RBCs during circulation in a heart–lung machine. A, Microvesicle concentration of 1‐week, 3‐weeks, and 5‐weeks‐old RBCs generated during circulation in the heart–lung machine (95% CI: 1‐week old: 2.4‐9.6 × 10^3^; 3‐weeks old: 1.2‐3.1 × 10^3^; 5‐weeks old: 1.5‐8.5 × 10^3^). For each storage period, RBCs of three different donors were pooled, as described in Materials and Methods; B, microvesicle concentration of plasma of five patients during extracorporeal circulation‐assisted cardiac surgery + confidence limits (95% CI). Microvesicles were isolated, quantitated, and characterized as described before (30). Abbreviations are as described in the legend to Figure 1. In panel A, the values of 1‐week‐old RBC derived microvesicles are significantly different from each other for all time points (*, *P* < .05)

During circulation in the stand‐alone extracorporeal circuit, the blood bank RBCs produced increasing numbers of microvesicles, especially after 3 hours (Figure 5A). In contrast, the microvesicle concentration in the patients’ blood was much lower and decreased after the first hour of circulation/surgery time (Figure 5B).

It has been suggested that storage‐associated susceptibility to oxidative stress may damage RBCs in an extracorporeal circulatory system.^10^ We observed an increase in methemoglobin in 1‐week‐old RBCs during circulation in the stand‐alone extracorporeal circuit and in the patients’ RBCs during surgery (Figure S2 in Supplemental Data), supporting this suggestion. The methemoglobin content was correlated with the microvesicle concentration in the patients’ blood (*r*, 0.639; *P* < .05; *N* = 17).

## 4 DISCUSSION AND CONCLUSION

During their passage through an extracorporeal circuit, RBCs are exposed to an environment that is very different from the circulation in vivo. The prolonged immersion of RBCs in a nonphysiological solution, interaction with artificial surfaces, and turbulence induce structural alterations that affect their biophysical, biochemical, and immunological properties.^22^ Extracorporeal circulation devices may be used in combination with blood transfusion and thus with RBCs that have undergone blood bank‐associated alterations in structure, metabolism, and function.^23^ Heart–lung machines meet the criteria for RBC integrity, but surgery involving extracorporeal circulation has side effects related to changes in RBC function and survival.^24^ Our present data show that the circulation of RBCs through a heart–lung machine induces physiologically relevant changes in the RBC structure that affect function and that are likely to decrease their survival. For RBCs from storage units, most changes showed a biphasic pattern, in which the early phase may represent the response of the fraction of the oldest, most vulnerable cells of the population. The second phase is likely to be dominated by the resulting shift toward an overall younger, more resilient RBC population. The duration of the first phase increased with RBC storage time, probably because the size of the most vulnerable fraction increases with aging in vitro, in this case with the time spent in the blood bank.^11^, ^18^ During surgery these changes were less pronounced, which may be attributed to the continuous physiological removal of dysfunctional RBCs. Mechanical stress in the heart–lung machine together with the surgery‐associated changes in the blood may induce changes in membrane organization that cause the observed changes in deformability and aggregation and increase in removal signals (Figures 3 and 4). The changes in aggregation behavior we observed for the RBCs of patients undergoing EC‐assisted surgery were observed for a relatively short period of time (Figure 3), but others have described much longer lasting effects.^4^

The changes in cell volume, osmotic fragility, and deformability indicate that changes in membrane organization that affect mechanisms involved in the transport of water and/or ions occur mainly in the first hours of circulation in an extracorporeal circuit.^7^ Later changes, especially the observed decrease in maximal deformability (Figure 3), are mainly due to a decrease in the surface area/volume ratio.^25^ The concomitant increase in microvesicle numbers (Figure 5) suggests that the latter is caused by a loss of membrane. The higher microvesicle concentrations observed in the experiments with the youngest RBCs (Figure 5) are probably due to the residual presence of reticulocytes with an aberrant vesiculation behavior, that are no longer present in the older concentrates.^21^ The differences between the stand‐alone extracorporeal circuit and the patient data are likely to be due to the fast removal of microvesicles from the patients’ circulation.^11^, ^20^

In order to reduce the need for transfusion, an autotransfusion system was used to wash and return perioperatively shed blood to the patient. However, with its suction and extensive blood‐air contact, this device can significantly contribute to RBC alterations. To reduce this interference, washed autotransfusion blood was returned after weaning from cardiopulmonary bypass and would, therefore, only affect the after circulation measurement (AC in the Figures). The total volume of returned autotransfusion blood included the residual blood from the heart–lung machine, further reducing the potential impact of autotransfusion‐altered RBC on the AC measurement.

Thus, our results show that circulation in an extracorporeal circuit has an impact on RBCs that extends well beyond hemolysis. The similarity of the data from our stand‐alone setup with those observed in the patients’ RBCs during extracorporeal bypass‐assisted surgery shows that in particular for the youngest storage units, circulation of RBCs in a stand‐alone device mimics many subtle alterations in the structure and function of the RBCs in vivo. This provides a biophysical, biochemical, and immunological system for the development and improvement of extracorporeal and other flow‐assist device technologies.^3^, ^26^ Also, these findings suggest that, when transfusion becomes necessary during surgery, the youngest available blood bank concentrates are preferred. Neonates, children or adults who depend on extracorporeal oxygenation and/or circulatory support for extended periods may be much more affected by the alterations in RBCs than observed in this study. These alterations are likely to contribute to various clinically significant observations during ECLS procedures, such as thrombotic events, decreasing thrombocyte and/or RBC levels necessitating blood product transfusions, and disseminated intravascular coagulation.^27^ Long‐term extracorporeal procedures might benefit the most from applying improved circuit components, using the RBC alterations investigated here as markers for biocompatibility.

## CONFLICT OF INTEREST

The authors declare that they have no conflicts of interest with the contents of this article.

## Supporting information

Fig S1

Fig S2
