# Supplementary figures and images for: The impact of circulation in a heart–lung machine on function and survival characteristics of red blood cells
Source: Artif Organs. 2020 Apr 3;44(8):892–9. doi: 10.1111/aor.13682 (PMC7496153; doi:10.1111/aor.13682)

**A**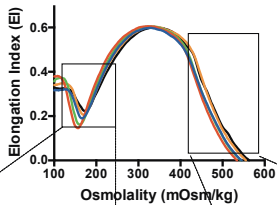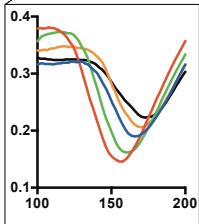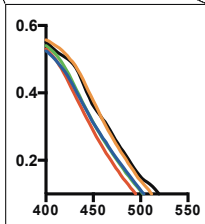

— BC  
— 30'  
— 1h  
— 2h  
— 4h

**B**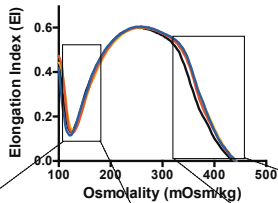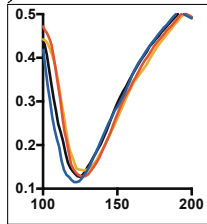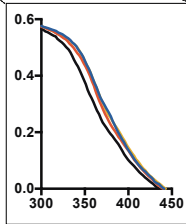

— BC  
— 30'  
— 2h  
— AC

Supplement: Supplementary file 1 — Fig S1 [file AOR-44-892-s001.pdf]

**A**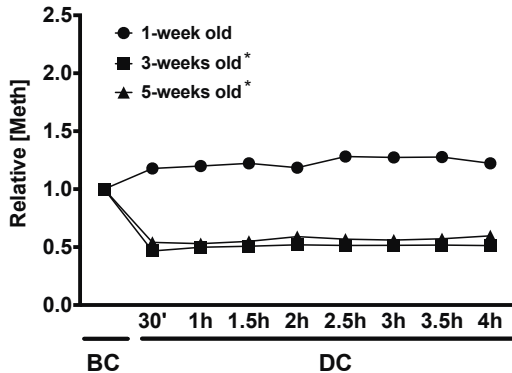**B**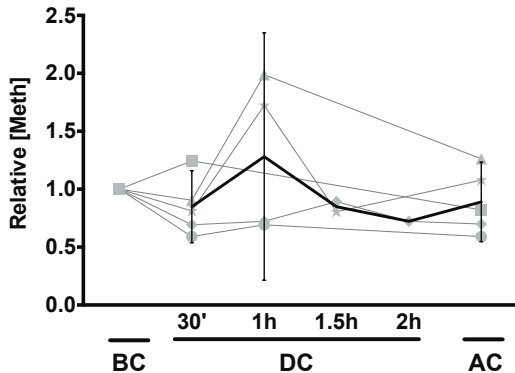

Supplement: Supplementary file 2 — Fig S2 [file AOR-44-892-s002.pdf]
